# Comparative genomic analysis unveiling the mutational landscape associated with premalignant lesions and early-stage gastric cardia cancer

**DOI:** 10.1097/MD.0000000000040332

**Published:** 2025-01-10

**Authors:** Guangda Wang, Liang Liu, Yang Zhao, Yan Lin, Limian Er

**Affiliations:** a Department of Computed Tomography and Magnetic Resonance, The Fourth Hospital of Hebei Medical University, Shijiazhuang, China; b Tumor Research Institute, The Fourth Hospital of Hebei Medical University, Shijiazhuang, China; c The Office of Academic Research, The Fourth Hospital of Hebei Medical University, Shijiazhuang, China; d Library, The Fourth Hospital of Hebei Medical University, Shijiazhuang, China; e Department of Endoscopy, The Fourth Hospital of Hebei Medical University, Shijiazhuang, China.

**Keywords:** early-stage GCA, EPHA2, mutation signature, NGS, premalignant

## Abstract

This study enrolled 10 patients diagnosed with premalignant lesions and early-stage gastric cardia adenocarcinoma (GCA), confirmed through endoscopic examination. These patients were subjected to next-generation sequencing (NGS) using a customized 1123-gene panel to identify genetic alterations and signaling pathways. The results were compared to stage IIB to IV GCA samples from the cancer genome atlas (TCGA) and a cohort of Hong Kong patients. The study provides insights into the molecular drivers of GCA progression, with potential therapeutic implications. A total of 10 patients diagnosed with premalignant and early-stage GCA were subjected to NGS targeted 1123-panal testing. Genetic alterations characteristics and signaling pathways were defined and analyzed. These findings were compared with the mutation features of stage IIB to IV GCA samples from the TCGA and another GCA cohort of HongKong patients (HK cohort). Additionally, therapeutic implications were also evaluated. In premalignant lesions and early-stage GCA, driver genes, such as TP53, ARIDA and LRP1B were found to have high mutation rates and showed no significantly different in driver gene mutation and tumor mutational burden with stage IIB to IV GCA in both the HK and TCGA-GCA cohorts. However, EPHA2 showed a significantly higher mutation rate in premalignant and early-stage GCA compared to IIB to IV GCA. The majority of 10 cancer-related signaling pathways were found to be activated in premalignant and early-stage GCA. Furthermore, 80% patients had corresponding potential therapeutic inhibitors based on molecular mutation results in our cohort. Certain mutational characteristics involved in the occurrence and progression of GCA are already present in premalignant lesions and early-stage GCA, which can be assessed and prevented through early molecular testing. Additionally, EPHA2 mutations are more common in premalignant lesions and early-stage GCA, which provided potential biomarkers for the diagnosis and detection of premalignant lesions and early-stage GCA.

## 1. Introduction

Gastric cancer (GC) is the fifth prevalent malignancy worldwide and ranks as the third most significant contributor to cancer-related mortality.^[1]^ It can be anatomically divided into gastric cardia adenocarcinoma (GCA) and gastric non-cardia adenocarcinoma (GCNA), with the latter having a significantly higher incidence in the vast majority of countries and regions compared to the former.^[2]^ However, in recent years, the incidence of GCA has been increasing year by year.^[3,4]^ The incidence of GCA in China is higher than that in Japan and most other regions, accounting for 23.2% of cases in resection of GC.^[5]^ GCA occurs in the narrow area of the proximal stomach below the gastroesophageal junction, and over 90% of patients have reached middle or late stages when they seek medical attention due to its hidden onset and obscure clinical symptoms and signs.^[6]^ The 5-year survival rate after standard treatment ranges from 16% to 32.3%.^[7]^

The premalignant gastric cardia lesions are classified into 2 levels: low-grade intraepithelial neoplasia and high-grade intraepithelial neoplasia (HGIN).^[8]^ Patients with HGIN have a significantly increased risk of developing to the GCA.^[9]^ Since the curable premalignant gastric cardia lesions are usually asymptomatic and hidden, most patients are already in the middle or late stages when they are clinically diagnosed.^[10]^ Early detection, early diagnosis, and early treatment are the key to improve the survival rate of patients with GCA and reducing their tumor burden.

Currently, endoscopic examination and biopsy are the main screening methods for high-risk areas of GCA in China. However, as the previous study have shown, the current plan exhibits a low positivity rate within the target population.^[11]^ The latest progress in next-generation sequencing (NGS) technology has significantly enhanced our comprehension and exploration of cancer genomics. In a study of the cancer genome atlas (TCGA), GC was categorized into 4 subtypes, including Epstein-Barr virus subtype, microsatellite instability (MSI) subtype, genome-stable (GS) subtype, and chromosomal instability (CIN) subtype.^[12]^ It was identified that GCA is a CIN subtype cancer similar to the Lauren intestinal-type, which is enriched in TP53 mutations and receptor tyrosine-kinase-RAS activation.^[13]^ The metabolism of nicotinamide mediated by NNMT was found to be associated with tumor initiation and inflammation-induced angiogenesis. Additionally, the expression level of NNMT was observed to gradually increase during the malignant progression of cardia adenocarcinoma and was significantly connected to poor prognosis.^[14]^ However, the mechanisms underlying the occurrence and progression of GCA are not yet clear.

In this study, we aim to investigate the molecular characteristics of mutation in premalignant and early-stage GCA through deep sequencing and explore the potential molecular factors associated with precancerous-cancer progression by comparing the mutation molecular features of precancerous and cancerous tissues.

## 2. Materials and methods

### 2.1. Patients and data

In this study, we enrolled 10 patients with premalignant gastric cardia lesions diagnosed by endoscopic examination for endoscopic submucosal dissection (ESD) or cardia mucosal lesion resection in the Fourth Hospital of Hebei Medical University (HB cohort) from March 2021 to March 2023. The sample size of 10 patients was determined based on the availability of patients meeting the inclusion criteria during the study period (March 2021 to March 2023) and was designed as an exploratory study to characterize genomic alterations in premalignant and early-stage GCA. Future studies with larger cohorts will be necessary to validate these findings and establish statistically robust conclusions. All samples were independently examined by 2 experienced pathologists. The patients were pathologically classified into HGIN, which belonged to premalignant gastric cardia lesions, and early-stage GCA. Postoperative samples were formalin-fixed paraffin-embedded (FFPE), and subjected to NGS-based 1123 panel gene sequencing. The 1123-gene sequencing panel was used to perform deep sequencing on samples from patients with GCA. This panel covers key driver genes such as TP53, ARID1A, LRP1B, and EPHA2, as well as genes involved in cancer-related signaling pathways. Signaling pathway analysis was performed using the R packages “maftools” and “clusterProfiler,” which were applied to identify the activation of cancer-related pathways, such as TP53, PTK-RAS, Wnt, and PI3K, across the patient cohort. The selection of genes and regions was based on relevance to GCA progression and curated from databases including COSMIC, dbSNP, and ClinVar. This panel enabled us to detect critical genetic alterations and signaling pathway activations related to the initiation and progression of GCA. Germline DNA from matched blood samples was used as a reference for somatic mutation identification. Mutations present in both tumor tissue and matched blood were excluded as potential germline variants, with the focus on somatic mutations unique to the tumor samples. Detail information of filtered somatic mutation were recorded for all 10 patients (Table S1, Supplemental Digital Content, http://links.lww.com/MD/O262). This cohort consisted of 4 females and 6 males, aged from 52 to 71 years, mean age was 62.40 ± 5.82 years. Additional information such as endoscopic results, preoperative pathology, postoperative pathology, smoking, family history of cancer disease, and BMI was also collected (Table 1).

**Table 1:** The clinical information of all the patients.

Patient ID	Gender	Age	Endoscopic examination	Preoperative pathology	Postoperative pathology	Smoking	Family history of cancer disease	BMI (kg/m^2^)
HB-1	Male	61	Premalignant gastric cardia lesions	Severe dysplasia	HGIN	Yes	Gastric cancer	≥28
HB-2	Male	64	Premalignant gastric cardia lesions	HGIN	HGIN	No	Gastric cancer	24.0 to 27.9
HB-3	Male	69	Premalignant gastric cardia lesions	HGIN	HGIN	No	Gastric cancer	24.0 to 27.9
HB-4	Female	58	Premalignant gastric cardia lesions	Severe dysplasia	HGIN	No	No	≥28
HB-5	Female	64	Premalignant gastric cardia lesions	HGIN	Early-stage GCA	No	No	≥28
HB-6	Female	61	Premalignant gastric cardia lesions	HGIN	Early-stage GCA	No	Unknow	24.0 to 27.9
HB-7	Female	67	Premalignant gastric cardia lesions	Severe dysplasia	Early-stage GCA	No	Unknow	18.5 to 23.9
HB-8	Male	71	Premalignant gastric cardia lesions	HGIN	Early-stage GCA	No	Lung cancer	≥28
HB-9	Male	52	Premalignant gastric cardia lesions	HGIN	Early-stage GCA	Yes	No	24.0 to 27.9
HB-10	Male	57	Premalignant gastric cardia lesions	HGIN	Early-stage GCA	No	Esophagus cancer	24.0 to 27.9

GCA = gastric cardia adenocarcinoma, HIGN = high-grade intraepithelial neoplasia.

To identify the mutation characteristics of HGIN and early-stage GCA, we collected whole-genome sequencing (WES) data of HongKong GCA cohort from Wang et al (HK cohort).^[15]^ Specifically, we focused on stage IIB to IV cases for the analysis, which showed different stages from our HB cohort. The detailed information of clinical characteristics and sequencing results can be found in Table S2 and S3, Supplemental Digital Content, http://links.lww.com/MD/O262, respectively. In addition, mutational information of 103 patients with GCA were obtained from TCGA through UCSC Xena (https://xenabrowser.net/datapages/).

### 2.2. DNA isolations and target NGS

In this study, genomic DNA was extracted from postoperative tissue samples and matched blood samples of 10 patients using the Maxwell RSC FFPE plus DNA kit (Promega, Cat no. AS1720) following the manufacturer’s instructions. Subsequently, 100 ng of gDNA was fragmented into 200 bp fragments using the Covaris E210 system (Covaris, MA, Inc.). NGS libraries were constructed using the KAPA HyperPrep Kit (Roche, Shanghai, China, 07962312001) and the Agilent SureSelect XT Kit (Agilent, G9702C). Sequencing was performed on the Illumina NovaSeq 6000 platform (USA), and BCL files were converted to FASTQ files using the Bcl2fastq conversion software. The clean reads were then aligned to the human reference genome (UCSC hg19) using the Burrows–Wheeler Aligner (BWA version 0.7.11) algorithm.

### 2.3. Data processing

Somatic mutations, including small insertions, deletions, and single nucleotide polymorphisms (SNPs), were called using the IndelRealigner module of the Genome Analysis Toolkit (GATK, version 3.6) and the VarScan software, using their default settings. The frequency threshold for common SNPs was set at 1%, using data from the 1000 Genomes Project and dbSNP databases. Mutations found in more than 1% of the population were filtered out. Additionally, synonymous mutations and those with fewer than 8 supporting reads were excluded from the analysis to ensure the robustness of the somatic mutation calling process and to minimize false-positive results. Annotation of all variants was performed using annotation filters, genes, and regions based on databases such as dbSNP (build 147), ClinVar, COSMIC (ver. 4), ANNOVAR, MutationTaster, CADD, and the 1000 Genomes Project. To identify SNVs and indels, the following filtering criteria were applied to candidate mutations: removal of mutations within introns, removal of mutations reported in more than 1% of the population in the 1000 Genomes Project (1000gau_2015all) or EXAC_all, filtering out common SNPs found in dbSNP (http://www.ncbi.nlm.nih.gov/SNP), exclusion of synonymous variants, and removal of mutations with <8 supporting reads.

### 2.4. Tumor mutation burden (TMB) and SNP analysis

TMB was calculated based on the number of all nonsynonymous mutations and the number of indexes per MB. Differences in TMB among HB, HK, and TCGA-GCA cohorts were analyzed using the R package “ggplot” [ggplot2: elegant graphics for data analysis]. The R packages “survival” [_modeling survival data: extending the Cox model_.] and “survminer” [survminer: drawing survival curves using “ggplot2”] were applied to analyze the correlation between TMB and prognosis.

The R package “circlize” [circlize implements and enhances circular visualization in R.] was used to show the SNP that occurred in each sample.

### 2.5. Amino acid changes

The 3-dimensional protein structure of the gene was screened by the PDB database (http://www.rcsb.org/), and PyMOL version 2.4.0 was used to predict the effect of gene mutations on the protein structure.

### 2.6. Mutational signature analysis

Cancer is an outcome of DNA mutagenesis process and can be inferred from somatic mutation signatures by analyzing the genome sequence. To detect the difference in signature between premalignant lesions and early-stage GCA samples, mutation signature detection base pair of 6 substitution subtypes: C > A, C > G, C > T, T > A, T > C, and T > G. The optimal number of clusters was selected according the parameters for the R package “NMF” [A flexible R package for nonnegative matrix factorization.]. In order to compare with known mutational signatures, we used the cosine similarity with the reference catalogue of somatic mutations in cancer (COSMIC) signatures.

### 2.7. Targeted inhibitors for gene mutations

Targeted inhibitors were mined based on mutation sites and displayed by drawing sankey diagram with the R package “ggalluvial” [ggalluvial: alluvial plots in “ggplot2”].

We identified targeted drugs for TP53 mutation based on the clinical data on cancer mutations from the clinical interpretation of variants in cancer (CIViC) database (https://civicdb.org/home). Download 3D protein structures of TP53 and etoposide from the PDB database (https://www.rcsb.org/). The 3D protein structure was processed by Pymol v2.4.0, including deleting water molecules and hydrogenation operations. The docking results of TP53 and etoposide were obtained by Autodock v4.2.6, and the optimal docking mode was screened according to binding energy and visualized by pymol v2.4.0.

### 2.8. Statistical analysis

Mutation correlation analysis was performed using the R package “maftools” [maftools: efficient and comprehensive analysis of somatic variants in cancer.]. For categorical variables, Wilcoxon test or Fisher exact test was used, and for continuous variables, a t-test was conducted. All reported *P*-values were 2-tailed, and *P* < .05 was considered statistically significant.

## 3. Results

### 3.1. Patient characteristics

Endoscopic examination is the primary diagnostic method for GCA in high-risk areas in China.^[16]^ In our study, 10 patients were diagnosed with premalignant gastric cardia lesions through endoscopic examination. ESD or cardia mucosal lesion resection of the premalignant gastric cardia lesions were performed followed by postoperative pathological examination, the detailed clinical information was shown in Table 1. Among these cases, 4 were consistent with the endoscopic findings and showed HGIN, indicating premalignant gastric cardia lesions. Two cases exhibited gastric cardia submucosal infiltration, while 4 cases had involvement of the mucosal muscle layer. In 6 cases, there were no lymph node metastasis observed in either the submucosal layer or the mucosal muscle layer, which classified as early-stage GCA.^[17]^ The accuracy rate of endoscopic examination was 40%, which is lower than previously reported rates 50%.^[18]^

Four patients had a family history of upper gastrointestinal disease, including GC and esophageal cancer, and 1 patient had a family history of other types of tumors. A recent meta-analysis demonstrated a positive correlation between body mass index (BMI) and the risk of GC, including GCA.^[19]^ In our study, the majority of patients (90%) had a BMI above the normal range (24–27.9 kg/m^2^), with 4 patients (40%) having a BMI greater than or equal to 28 kg/m^2^, indicating obesity^[20]^ (Table 1). The relationship between clinicopathological features and mutation characteristic was shown in Figure S1A, Supplemental Digital Content, http://links.lww.com/MD/O261.

### 3.2. Somatic alterations in HGIN and early-stage GCA

**Figure 1.**
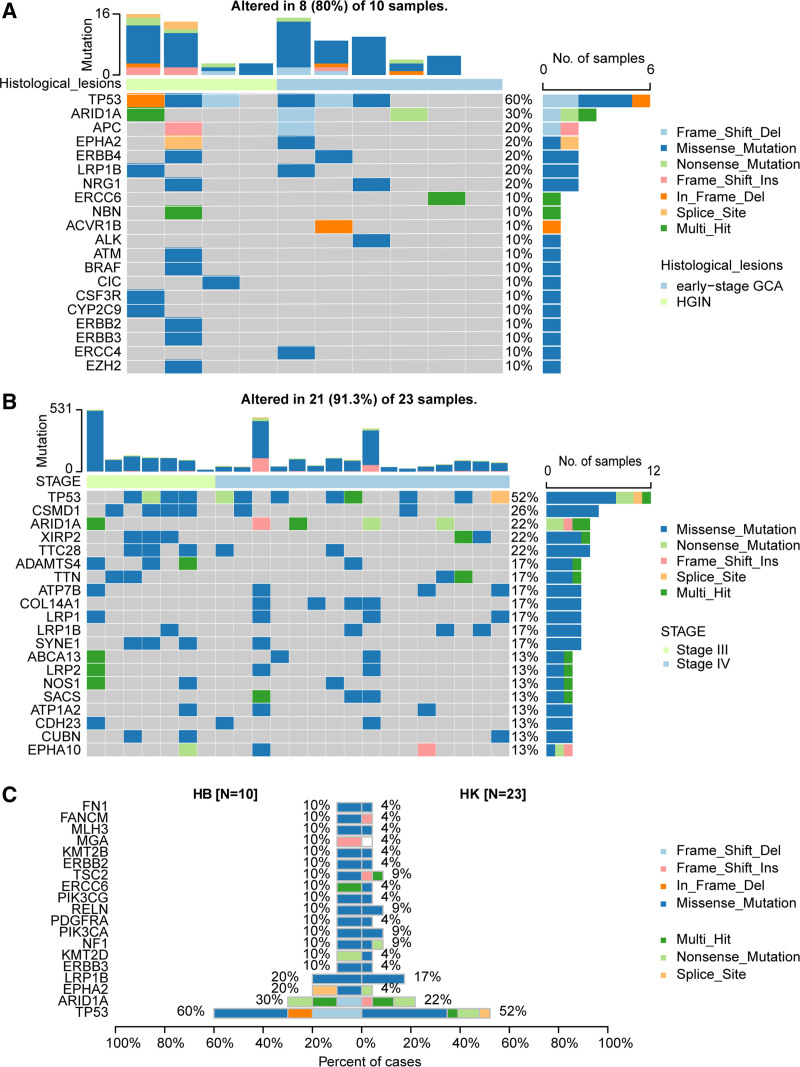
The somatic mutation features of HGIN and early-stage GCA, and IIB to IV GCA. (A) The genomic landscape of HGIN and early-stage GCA in HB cohort. (B) The genomic landscape of IIB to IV GCA patients in HK cohort. (C) Comparison of gene mutation rates between HGIN and early-stage GCA, and IIB to IV stage GCA. GCA = gastric cardia adenocarcinoma, HIGN = high-grade intraepithelial neoplasia.

We performed NGS-based 1123 panel gene sequencing on samples from 10 patients, the average coverage depth of tissue DNA was 3000x. Silent mutations, and unknown mutations in somatic mutations were excluded after screening. `In a total of 79 somatic genomic alterations from 65 genes were detected in 4 HGIN samples and 6 early-stage GCA samples. Table S1, Supplemental Digital Content, http://links.lww.com/MD/O262 showed the details of the mutation data for each patient, SNP variant was the majority type of the somatic alterations, and the characteristics of each patients of SNP variant are presented in Figure S1B, Supplemental Digital Content, http://links.lww.com/MD/O261. The detail of mutation profiles of the top 10 mutated genes in the 10 patients are shown in Table 2. The most frequently mutated gene was *TP53* (60%) (Fig. 1A), which is consistent with the findings in the HK cohort (Fig. 1B) and TCGA (Fig. S2A, Supplemental Digital Content, http://links.lww.com/MD/O261) on stage IIB to IV GCA. Other frequently mutated genes in HB cohort included ARIDA (30%), APC (20%), EPHA2 (20%), and ERBB4 (20%). One patient with HGIN had mutations in both ERBB4 and NGR1, as well as other members of the EGFR family (ERBB2, ERBB3). None of the 1123 mutated genes detected in our study were found in 1 early-stage GCA patient (Fig. 1A). In stage IIB to IV GCA samples from the HK cohort, the top 5 mutated genes with the highest mutation frequencies were TP53, CSMD1, ARID1A, TTC28, and XIRP2 (Fig. 1B). The concordance of driver mutations such as TP53, ARID1A, LRP1B, PIK3CA, and ERBB4 between the samples from HB cohort and HK cohort were examined. TP53, ARID1A, and LRP1B had relatively high mutation frequencies in both cohorts. Except for EPHA2, which had a higher mutation frequency in HGIN and early-stage GCA than in IIB to IV GCA in HK cohort, there were no significant differences in mutation frequencies of other genes between the 2 cohorts (Fig. 1C). There were no significant differences in mutation frequencies of TP53, ARID1A, and LRP1B between the HB patients and IIB to IV patients in TCGA-GCA. However, the PHA2 mutations were not detected in stage IIB to IV GCA in TCGA cohort (Fig. S2B, Supplemental Digital Content, http://links.lww.com/MD/O261). The mutation rates of TP53, ARID1A, and LRP1B genes had not significant differences across different stages in TCGA-GCA cohort, although the mutation of EPHA2 only found in patients of IIA stage. (Fig. S2C–F, Supplemental Digital Content, http://links.lww.com/MD/O261).

**Table 2:** Mutation characteristic of the patients.

Patient ID	postoperative pathology	Gene	Transcript	Chromosome	exon	Base change	AA change	Effect
HB-1	HGIN	ARID1A	NM_006015	chr1	exon1	c.317_318insCGCGGGC	p.P109Rfs*4	Frame_Shift_Ins
HB-1	HGIN	ARID1A	NM_006015	chr1	exon20	c.5221C > T	p.Q1741X	Nonsense_Mutation
HB-1	HGIN	LRP1B	NM_018557	chr2	exon45	c.7503C > A	p.N2501K	Missense_Mutation
HB-1	HGIN	TP53	NM_001126115	chr17	exon3	c.358_360del	p.L120del	In_Frame_Del
HB-2	HGIN	APC	NM_001127511	chr5	exon14	c.4646_4647insAGATG	p.D1551Efs*9	Frame_Shift_Ins
HB-2	HGIN	ERBB2	NM_001289937	chr17	exon8	c.937C > A	p.L313I	Missense_Mutation
HB-2	HGIN	ERBB3	NM_001982	chr12	exon23	c.2833G > A	p.V945I	Missense_Mutation
HB-2	HGIN	ERBB4	NM_001042599	chr2	exon27	c.3770G > A	p.R1257Q	Missense_Mutation
HB-2	HGIN	NRG1	NM_001322197	chr8	exon8	c.998A > C	p.K333T	Missense_Mutation
HB-2	HGIN	TP53	NM_001126115	chr17	exon3	c.347G > A	p.R116Q	Missense_Mutation
HB-3	HGIN	TP53	NM_001126115	chr17	exon3	c.384delC	p.S129Vfs*84	Frame_Shift_Del
HB-5	early-stage GCA	APC	NM_001127511	chr5	exon14	c.4599_4600del	p.E1534Gfs*6	Frame_Shift_Del
HB-5	early-stage GCA	ARID1A	NM_006015	chr1	exon1	c.275_285del	p.P94Afs*13	Frame_Shift_Del
HB-5	early-stage GCA	EPHA2	NM_001329090	chr1	exon13	c.2284C > T	p.R762W	Missense_Mutation
HB-5	early-stage GCA	LRP1B	NM_018557	chr2	exon63	c.10004A > T	p.K3335I	Missense_Mutation
HB-5	early-stage GCA	TP53	NM_001126115	chr17	exon2	c.218A > G	p.Y73C	Missense_Mutation
HB-6	early-stage GCA	ERBB4	NM_001042599	chr2	exon8	c.958A > C	p.I320L	Missense_Mutation
HB-6	early-stage GCA	TP53	NM_001126115	chr17	exon4	c.484delG	p.E162Sfs*51	Frame_Shift_Del
HB-7	early-stage GCA	ALK	NM_004304	chr2	exon1	c.337G > A	p.G113S	Missense_Mutation
HB-7	early-stage GCA	NRG1	NM_013962	chr8	exon1	c.67G > A	p.A23T	Missense_Mutation
HB-7	early-stage GCA	TP53	NM_001126115	chr17	exon1	c.38T > G	p.L13R	Missense_Mutation
HB-8	early-stage GCA	ARID1A	NM_006015	chr1	exon8	c.2469C > G	p.Y823X	Nonsense_Mutation
HB-9	early-stage GCA	ERCC6	NM_000124	chr10	exon5	c.1390C > T	p.R464W	Missense_Mutation
HB-9	early-stage GCA	ERCC6	NM_170753	chr10	exon2	c.787A > G	p.N263D	Missense_Mutation

GCA = gastric cardia adenocarcinoma, HIGN = high-grade intraepithelial neoplasia.

**Figure 2.**
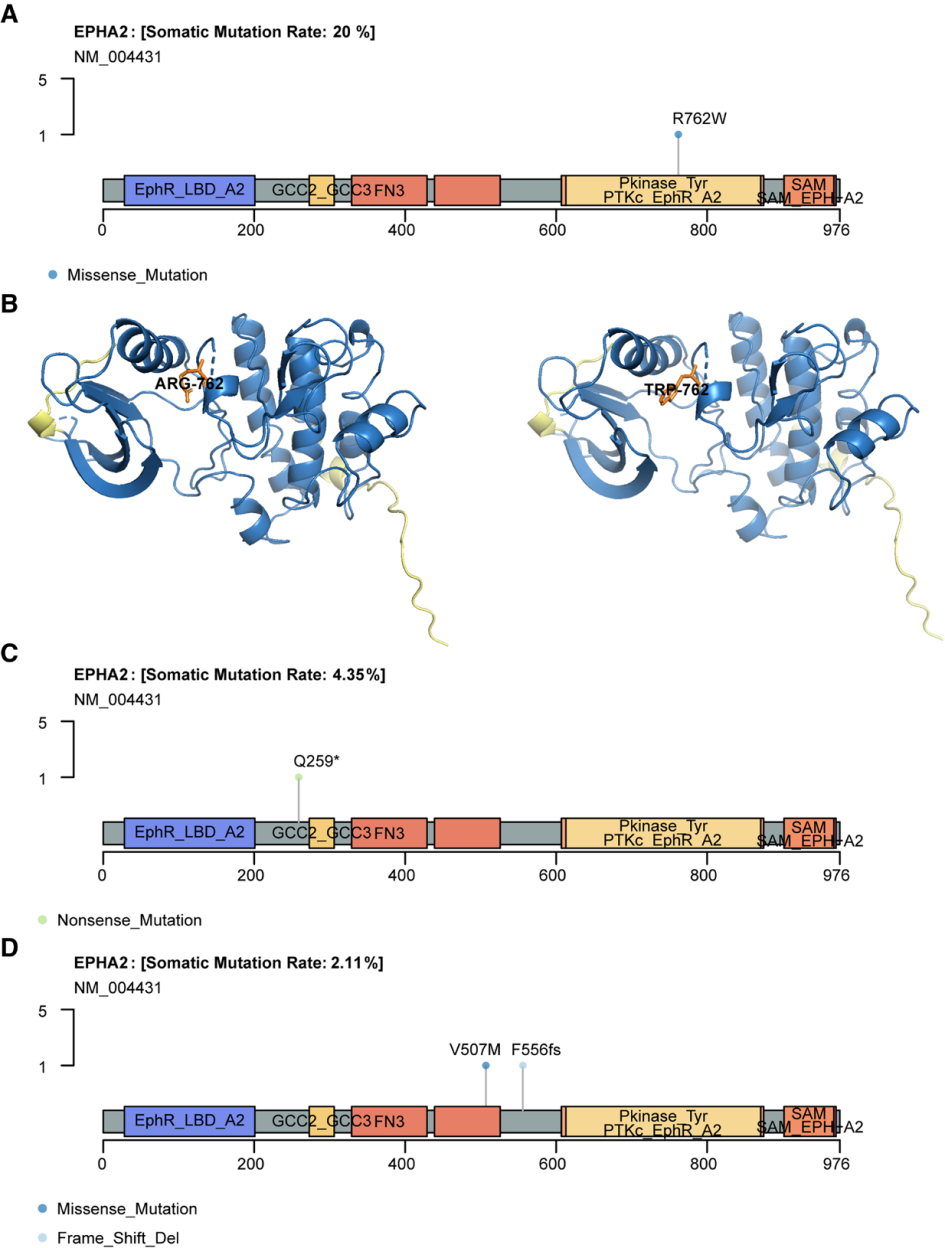
The mutation sites of EPHA2. (A) Lollipops plot shows informative mutations for EPHA2 in HB cohort. (B) The 3D structures of EPHA2 protein and somatic site. (C) Lollipops plot shows informative mutations for EPHA2 in IIB to IV GCA in HK cohort. (D) Lollipops plot shows informative mutations for EPHA2 in IIA GCA in TCGA cohort. X-axis labels for domain start and stop positions and exact marker locations are clearly displayed for mutation types. GCA = gastric cardia adenocarcinoma, TCGA = the cancer genome atlas.

Interestingly, in the 10 patients with HGIN and early-stage GCA from HB cohort, 2 cases had somatic variations in EPHA2, with mutation frequencies higher than those in the HK and TCGA cohorts of stage IIB to IV GCA, at 4% and 0%, respectively. Detailed analysis showed that the mutation of EPHA2 in HB cohort cases occurred in the tyrosine-kinase domain (R762W) (Fig. 2A), Figure 2B showed 3-dimensional protein structure and mutated site of the EPHA2. While in the HK cohort, the mutation in EPHA2 was at Q259 (Fig. 2C). However, no EPHA2 mutations were detected in the TCGA-GCA IIB to IV samples, and in the TCGA-GCA samples staged as I–IIA, 2 patients (2/37) had EPHA2 mutations, which were not in the kinase domain (Fig. 2D). These results suggest that EPHA2 is more commonly found in premalignant gastric cardia lesions and early-stage GCA, and it has the potential to serve as a biomarker for early-stage GCA identification.

### 3.3. Tumor mutational burden (TMB) analysis

TMB refers to the number of somatic mutations per megabase (Mb) in the sequenced cancer genome, including point mutations and insertions/deletions. TMB serves as a marker indicating the quantity of genetic alterations within a tumor sample, and it has significant implications in cancer research.

**Figure 3.**
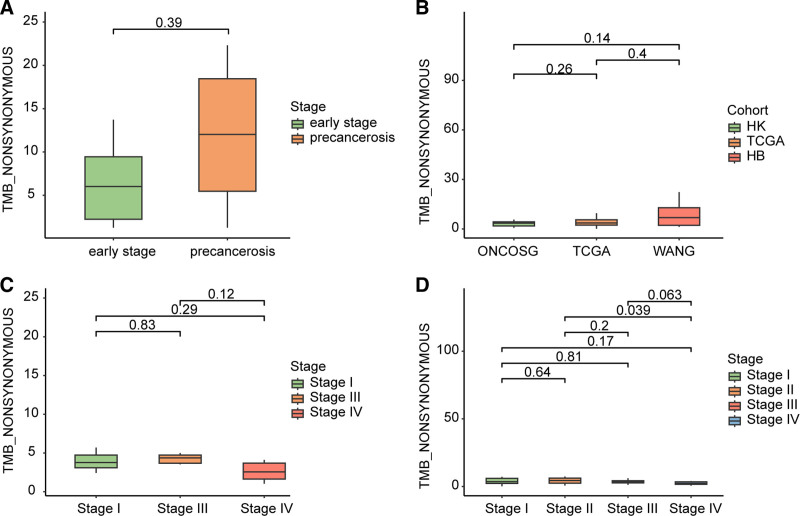
Box plots for TMB features. (A) Comparison of TMB between HGIN and early-stage GCA. (B) Comparison of TMB among HB cohort, HK cohort, and TCGA cohort. (C and D) The TMB profiling among different stages of GCA cohorts: (C) in HK cohort, (D) in TCGA cohort. GCA = gastric cardia adenocarcinoma, HIGN = high-grade intraepithelial neoplasia, TCGA = the cancer genome atlas, TMB = tumor mutational burden.

In our study, we calculated the TMB values for 10 patients, with a median TMB of 6.87 mutations per Mb (range, 1.26–22.32 mutations per Mb). The median TMB for HGIN was 12.02 (range, 1.26–22.32 mutations per Mb), and for early-stage GCA was 6.02 (range, 1.26–13.73 mutations per Mb).There was no statistically significant difference observed in the TMB between 2 specific groups (*P* = .39) (Fig. 3A). Additionally, when comparing the TMB of GC precursor lesions, such as HGIN and early-stage GCA in HB cohort, with advanced-stage GCA (IIB–IV) in cohorts from HK, and TCGA-GCA, no significant difference in TMB was found (Fig. 3B). These findings also showed that TMB does not significantly vary among different stages of GCA, regardless of the cohort being analyzed, including HK and TCGA-GCA cohort (Fig. 3C and D). Furthermore, no significantly association was found between overall survival (OS) and TMB subgroup (Fig. S3, Supplemental Digital Content, http://links.lww.com/MD/O261).

### 3.4. Mutation signatures

**Figure 4.**
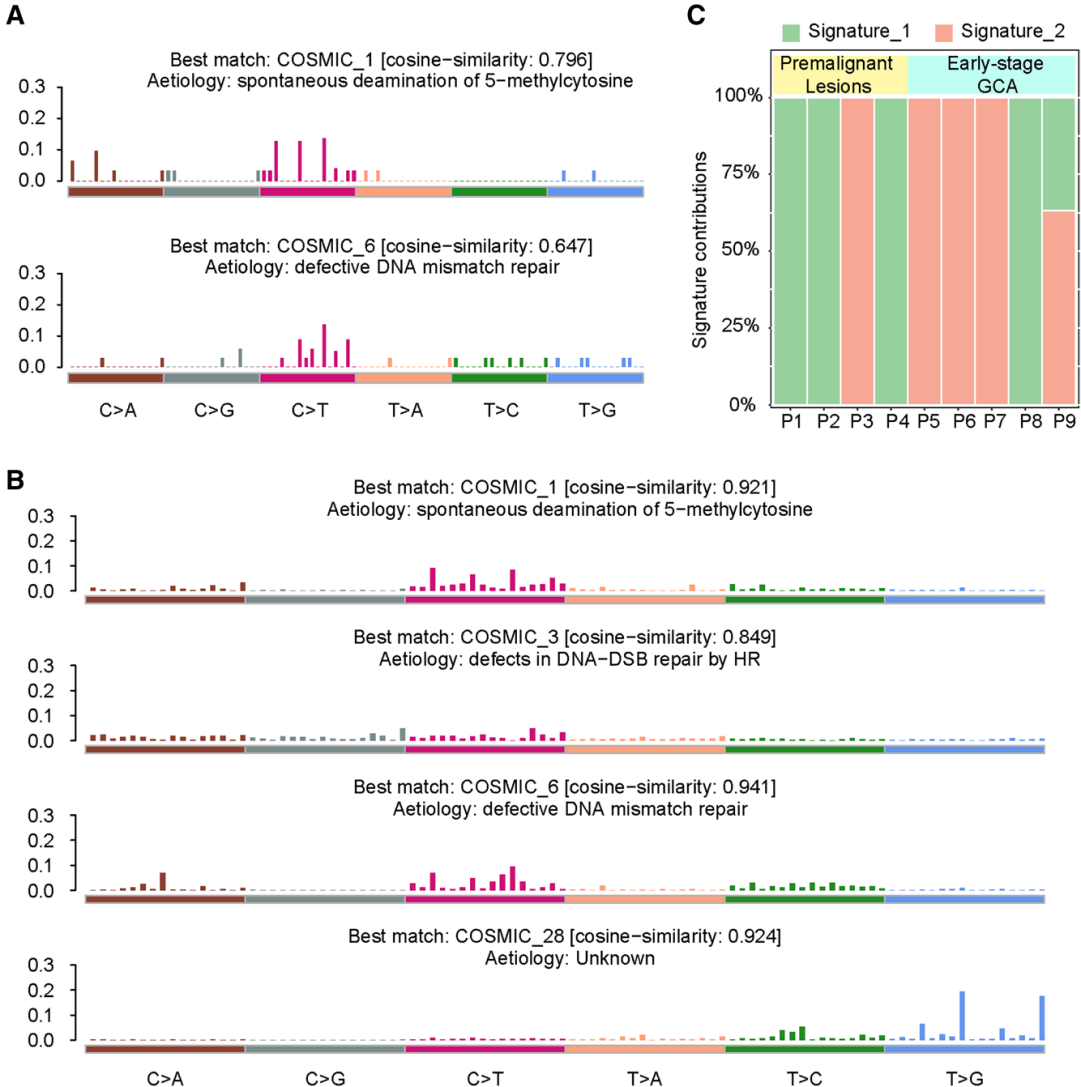
Profiles of mutational signatures in LGIN and early-stage GCA, and IIB to IV GCA. (A) The mutation signature of HB cohort and cosine similarity to COSMIC signatures. (B) The mutation signature of HK cohort and cosine similarity to COSMIC signatures. (C) Relative compositions of mutation signatures in HB cohort samples. Each column represents a patient. COSMIC = catalogue of somatic mutations in cancer, GCA = gastric cardia adenocarcinoma, LGIN = low-grade intraepithelial neoplasia.

The signatures of mutations are patterns of single nucleotide variations in somatic cells, which can reflect mutational processes.^[21]^ Using the ‘deconstructSigs’ R package, we calculated the contribution of each mutation feature to the 10 samples in the HB cohort. Our study identified 2 features, signature1 and signature2, that characterize the progression of HGIN and early-stage GCA (Fig. 4A). We evaluated the 2 signatures in the HB cohort using mutation signature from COSMIC database. Signature 1 matched COSMIC-1 (spontaneous deamination of 5-methylcytosine) with a cosine similarity of 0.796. Signature2 matched COSMIC-6 (defective DNA mismatch repair) with a cosine similarity of 0.674 (Fig. 4A, Fig. S4A, Supplemental Digital Content, http://links.lww.com/MD/O261). Based on the mutation patterns, signature1 is characterized by C > T and C > A mutations, while signature2 is primarily characterized by C > T mutations. Unlike signature1, the proportion of T > C mutations is elevated in signature2. Additionally, we identified 4 signatures in the IIB to IV GCA mutations in the HK cohort. Signature1 and signature3 matched COSMIC-1 and COSMIC-6, respectively. The other 2 signatures, signature2 and signature4, matched COSMIC-3 (defects in DNA-DSB repair by HR) and COSMIC-28 (unknown), respectively (Fig. 4B, Fig. S4B, Supplemental Digital Content, http://links.lww.com/MD/O261). We conducted a detailed analysis of the mutation signatures in 9 patients from the HB cohort, and the results showed that most samples contained only 1 signature. Signature1 was more frequently observed in HGIN (75%, 3/4), while most early-stage GCA samples contained only signature2 (60%, 3/5) (Fig. 4C).

### 3.5. Germline mutations

**Figure 5.**
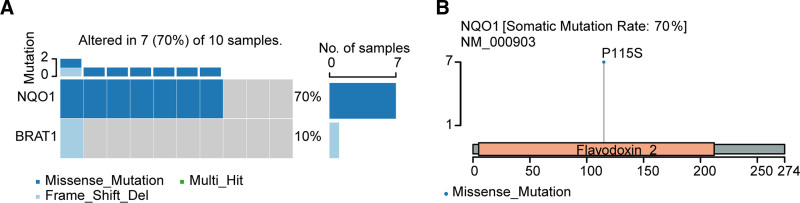
The germline mutation of HGIN and early-stage GCA. (A) The cancer-related germline mutations of HGIN and early-stage GCA in HB cohort. (B) Lollipops plot shows informative mutations for NQO1 in HB cohort. GCA = gastric cardia adenocarcinoma, HGIN = high-grade intraepithelial neoplasia.

Among 10 patients with HGIN and early-stage GCA, among the tumor-associated genes, 7 cases were found to have germline mutations in the NAD(P)H:quinone oxidoreductase 1 (NQO1). One case of HGIN was found to have 2 tumor-associated genes with germline mutations, including NQO1 and BRAT1 (Fig. 5A). All cases with germline mutations in NQO1 had the P115S mutation, which is a missense mutation (Fig. 5B). NQO1 is a metabolic enzyme involved in the detoxification of many exogenous compounds.^[22]^

### 3.6. Cancer-related pathway analysis

**Figure 6.**
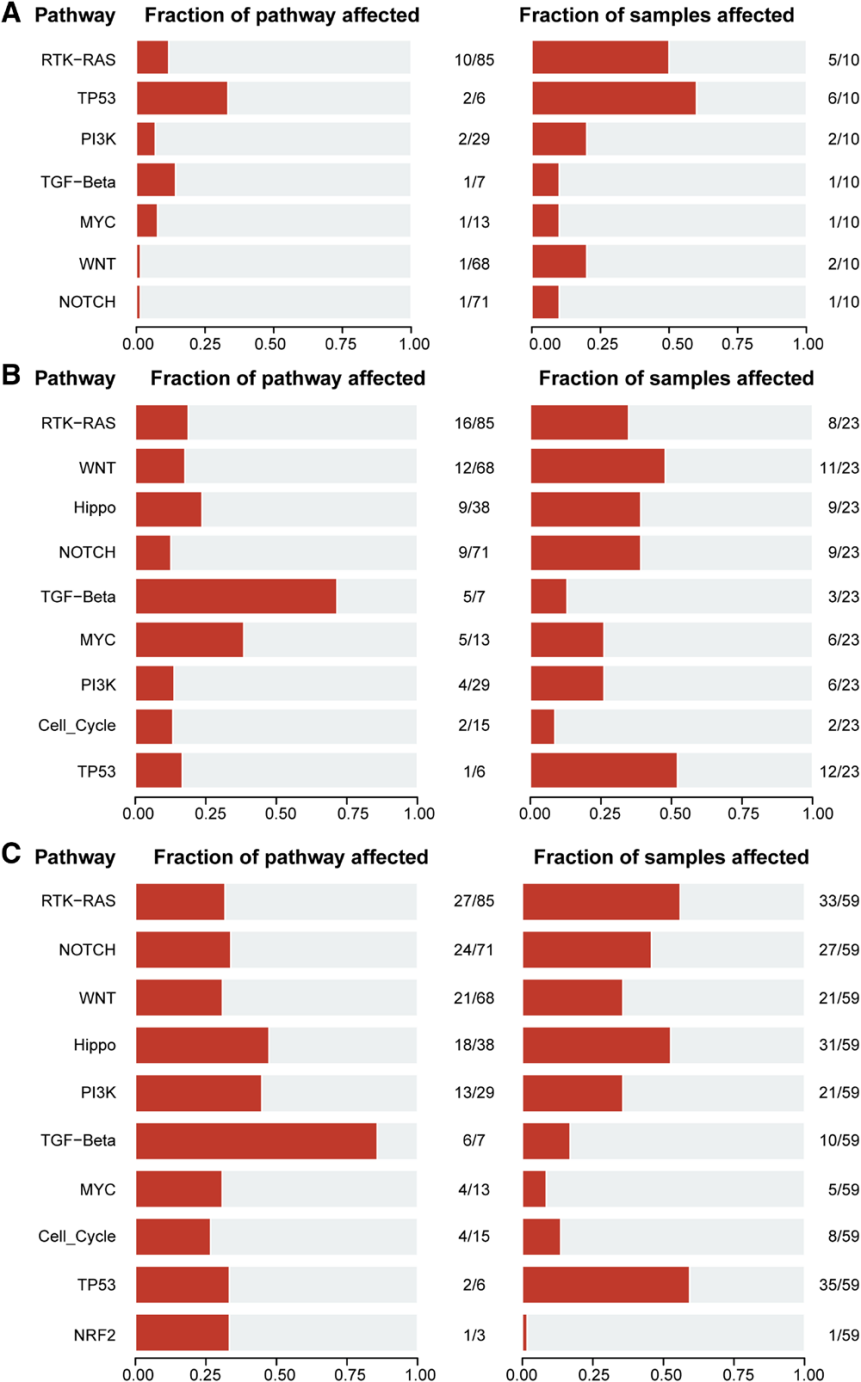
Canonical pathway analysis in HGIN and GCA. (A–C) The frequently mutated pathway of 10 cancer-related canonical pathways in different cohort samples: (A) HB cohort samples, (B) IIB to IV GCA samples in HK cohort, (C) IIB to IV GCA samples in TCGA cohort. GCA = gastric cardia adenocarcinoma, HGIN = high-grade intraepithelial neoplasia, TCGA = the cancer genome atlas.

According to the statistical analysis of 10 cancer-related signaling pathways in the HB cohort, multiple typical oncogenic signaling pathways were found to be activated. The most frequently mutated pathway was the TP53 signaling pathway, with 6 out of 10 samples (60%) having mutations in 2 out of 6 (40%) TP53 pathway-related genes. The PTK-RAS signaling pathway was the next most affected, with 5 samples (50%) exhibiting mutations, followed by the Wnt and PI3K signaling pathways, which had mutations in 2 samples each (Fig. 6A). In the IIB to IV stage GCA from HK and TCGA cohorts, the most commonly affected pathway was the P53 pathway, with 35 (59%) and 12 (52%) cases, respectively (Fig. 6B and C).

In addition to the aforementioned PTK-RAS, Wnt, and PI3K signaling pathways, significantly enriched mutations were observed in the Hippo signaling pathway in patients with IIB to IV GCA in HK and TCGA cohorts (Fig. 6B and C). However, there were no Hippo pathway genes mutated in the HB cohort. The Hippo pathway is an important signaling cascade that regulates biological processes such as cell proliferation, apoptosis, and tissue repair. Under normal circumstances, the Hippo pathway suppresses tumor development and maintains normal tissue growth and homeostasis. However, abnormal activation or inhibition of the Hippo pathway can disrupt cell proliferation and survival, thereby promoting tumor development.^[23]^

### 3.7. Therapeutic implications of somatic alterations

**Figure 7.**
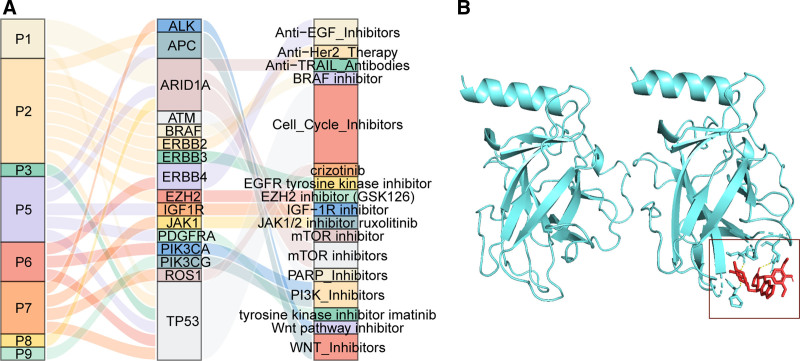
Therapeutic implications of somatic alterations of each patient in the Fourth Hospital of Hebei Medical University (HB) cohort. (A) Landscape of somatic altered genes and their corresponding putative therapeutic implications of each patient in HB cohort. (B) Simulations of the binding interactions between etoposide and the TP53 protein.

Among the 10 patients, 8 (80%) had at least 1 potential therapeutic inhibitor-associated genetic alteration. Six patients had TP53 mutations, suggesting potential sensitivity to cell cycle inhibitors (Fig. 7A). Targeted therapy directed towards tumor-specific mutations provides personalized treatment strategies for patients. Based on information obtained from the CIViC database, we identified Etoposide as a potential targeted drug for TP53 mutations in stomach cancer. Additionally, we performed simulations of the binding interactions between etoposide and the TP53 protein complex to investigate its binding characteristics (Fig. 7B). Other commonly observed genetic mutations with potential therapeutic implications included APC, ARID1A, ERBB4, BRAF, and PIK3CA, corresponding to WNT inhibitors, mTOR inhibitors, Anti-EGF inhibitors, BRAF inhibitors, and PI3K inhibitors, respectively.

## 4. Discussion

Due to the unclear exact etiology of GCA, primary prevention targeting the etiology is difficult to carry out. The main measures for comprehensive prevention and control of GCA currently focus on early detection, diagnosis, and treatment, as well as screening and early diagnosis of GCA and its premalignant gastric cardia lesions. Endoscopic examination is the most widely used screening method for GCA in high-risk areas in China.^[24]^ However, there are problems with low screening efficiency and low positive rate in endoscopic examination.^[25]^ GCA is a subject of insufficient research. The pathogenesis, molecular changes in the premalignant gastric cardia lesions and early-stage of carcinogenesis, and related risk factors have not been well described.^[26]^ Increased gene alterations often lead to the development of cancer. In recent years, the continuous development of sequencing technology has enabled the identification of a series of driver genes for cancer occurrence, providing a better understanding of the process of cancer initiation and development. In recent years, some reports have studied the molecular mechanisms and genomic variations in GC. TP53 is the most common mutation found in gastric and esophageal cancer,^[27]^ and abnormal function of the p53 gene leads to uncontrolled cell cycle and abnormal cell proliferation.^[28]^ Besides TP53, LRP1B and ARID1A are the most common mutated genes in GC and play a crucial role in the occurrence and development of GC.^[29,30]^ One study suggested that TP53 mutations occur earliest in GC and may drive the onset of GC. LRP1B mutations in GC may be associated with the progression of GC.^[31]^ Consistent with the above research, in the premalignant and early-stage GCA, and stage IIB to IV GCA, the mutation rates of TP53, ARID1A, and LRP1B are relatively high, and there is no significant difference between the early and late stages. This suggests that hotspot mutations of TP53, LRP1B, and ARID1A, as potential cancer driver factors, occur earlier in the process of GCA initiation and are significantly enriched in cancer-related signaling pathways such as TP53, PTK-RAS, Wnt, and PI3K. Therefore, early detection and prevention can be conducted before the occurrence and development of GCA.

Ephrin type-A receptor 2 (EPHA2) is overexpressed in multiple tumors and is associated with poor prognosis, increased metastatic potential, and reduced survival rates in cancer patients.^[32,33]^ In this study, we identified a novel EPHA2 gene mutation (p.R762W) in the kinase domain. Two key sites in the kinase domain of EPHA2 exhibit opposite effects: Y772, when activated, inhibits cancer cell survival and migration, while S897 activation promotes cancer progression.^[34,35]^ Studies have shown that mutations A859D and T647M in the EPHA2 kinase domain can suppress the phosphorylation of Y772, significantly increasing tumor cell proliferation.^[36]^ The novel mutation site identified in HGIN and early-stage GCA in this study warrants further investigation into its impact on Y772 and S897, in order to gain a deeper understanding of the characteristics of HGIN and early-stage GCA and provide guidance for early detection and treatment of GCA.

ERBB4 binds to NRG1, forming homodimers and heterodimers, which subsequently activate kinase activity. In a Chinese study related to GC, it was found that 3 patients had simultaneous mutations in ERBB4 and NRG1, accounting for approximately 1%, and mutations in ERBBs and NRGs were found to be mutually exclusive in TCGA GC-related research.^[37]^ Among our cohort of 10 patients with HGIN and early-stage GCA, 1 patient had concurrent mutations in ERBB4 and NRG1, accounting for 10%. Whether this phenomenon is more pronounced in the Chinese population in the context of gastric and esophageal cancer research remains to be explored in future studies. Nonetheless, ERBB signal inhibitors have demonstrated promising efficacy in clinical trials across multiple tumor types, including GC and esophageal cancer.^[38,39]^ Further research and clinical investigation are warranted to elucidate their exact role and therapeutic efficacy in the management of this specific subtype of cancer. Additionally, our study further revealed that at least 1 gene alteration with targeted therapeutic significance exists in the majority of HGIN and early-stage GCA. Furthermore, in GC-related research, including in vitro and in vivo studies, these inhibitors have demonstrated antitumor activity when used alone or in combination.^[40–46]^

The polymorphism P187S in human NQO1 eliminates the enzymatic activity of NQO1 by greatly reducing its binding affinity to FAD, which is associated with an increased risk of various cancers, including prostate cancer, adult brain tumors, and colorectal adenoma.^[47–49]^ Furthermore, it serves as an important prognostic factor for breast cancer.^[50]^ In this study, a novel germline mutation, P115S, was identified in 6 patients (60%). The simultaneous occurrence of this missense mutation suggests that NQO1 P115S is a critical mutational hotspot in early esophageal cancer. This finding provides insights for future biomarker development and targeted therapies for esophageal cancer.

TMB is an important parameter for predicting the sensitivity to immune checkpoint inhibitors. In the study by Lee et al, the TMB in GC was significantly higher than that in gastric adenoma (GA), suggesting that TMB can serve as a marker for the progression from GA to GC.^[51]^ There have been reported that TMB-H was associated with longer survival and improved progression-free survival.^[52]^ However, in our study, there was no significant difference in survival between TMB-H and TMB-L samples from the TCGA-GCA cohort. Additionally, there was no significant difference in TMB between HGIN lesions and early-stage GCA, as well as between early-stage and advanced-stage GCA. The differences observed between GC and GCA may contribute to these results. Furthermore, the limited size of the analyzed cohort and variations in immunotherapy may have contributed to these inconsistent findings. Further analysis using larger cohorts of GCA samples is needed to gain a more comprehensive understanding of the mechanisms underlying the occurrence and progression of GCA.

Mutation signatures can reflect characteristic changes in the mutation process and DNA damage and repair in somatic cells.^[31]^ We identified 2 mutation signatures in HGIN lesions and early-stage GCA, referred to as signature1 and signature 2. The identified mutation signatures provide important insights into the molecular mechanisms underlying the progression of premalignant lesions to early-stage GCA. Signature 1, which is characterized by C > T and C > A mutations, is consistent with COSMIC Signature 1, indicating spontaneous deamination of 5-methylcytosine. This signature is commonly associated with aging-related mutagenesis and may represent the accumulation of mutations during the premalignant stage, prior to significant genomic instability. The presence of this signature in premalignant lesions suggests that mutational processes related to DNA methylation changes might be an early driver in GCA carcinogenesis In contrast, Signature 2 is associated with C > T mutations and matches COSMIC Signature 6, which is linked to defective DNA mismatch repair (MMR). This signature is typically found in cancers with high levels of microsatellite instability (MSI) and reflects a later stage of genomic instability. The detection of Signature 2 in early-stage GCA samples indicates that the acquisition of MMR defects may play a role in the progression from premalignant lesions to early malignancy. These findings suggest that while Signature 1 may serve as an early biomarker for identifying high-risk premalignant lesions, Signature 2 could be indicative of more advanced genomic alterations that promote the transition to early-stage GCA. Both signatures showed a relatively high proportion of C > T mutations, particularly in signature1. Previous studies have indicated that C > T mutations may represent mutations occurring prior to the emergence of tumor-initiating cells and they are consistently enriched with common mutations.^[53]^ In our study, the majority of HGIN lesions samples contained only signature1.

In this study, we identified several potential therapeutic inhibitors targeting the frequently mutated genes TP53, ARID1A, and EPHA2 in premalignant and early-stage GCA. For instance, Cell Cycle Inhibitors such as Etoposide have shown efficacy in cancers with TP53 mutations.^[54]^ Additionally, EPHA2-targeted therapies, which include small-molecule inhibitors and monoclonal antibodies, are currently being explored in clinical trials for other cancers, such as lung and esophageal cancer. Although EPHA2 inhibitors have not yet been tested specifically in GCA, their effectiveness in related cancers suggests potential applicability in treating GCA with EPHA2 mutations.^[55]^

Moreover, inhibitors targeting ARID1A deficiency, such as mTOR inhibitors and PI3K inhibitors, have demonstrated preclinical activity in GC models and are being evaluated in clinical trials. Given the frequent occurrence of ARID1A mutations in our cohort, these inhibitors could be considered as a potential therapeutic option for early-stage GCA patients with similar genetic alterations. Future clinical studies focusing on these inhibitors in the context of GCA could pave the way for more personalized treatment strategies and improve patient outcomes.^[56]^

Overall, the integration of these findings into clinical practice would require further validation in larger cohorts and clinical trials. Nevertheless, our study provides a foundation for exploring these inhibitors as part of a targeted therapeutic strategy for GCA, particularly in the early stages of the disease.

This study focuses on premalignant gastric cardia lesions and early-stage GCA which in a relatively favorable prognosis, so a majority of patients did not undergo regular follow-up endoscopy examinations, resulting in a lack of prognostic information. Additionally, the limited sample size is due to the difficulty in premalignant gastric cardia lesions and early-stage GCA. Therefore, future studies should focus on increasing the sample size to overcome these limitations.

## 5. Conclusion

Driver genes in GC, such as TP53, ARIDA, and LRP1B, have been found to be mutated in premalignant gastric cardia lesions and early-stage GCA, and the mutation rates of these genes are not significantly different from those observed in stage IIB to IV GCA. Interestingly, there is no significant difference in TMB between HGIN and early-stage GCA, nor compared to stage IIB to IV cardia cancer. This suggests that the gene mutation signature is already present in the early stages of cardia cancer, even before the appearance of clear malignant characteristics on endoscopy. Furthermore, EPHA2 mutations are more commonly observed in HGIN and early-stage GCA. This finding provides a potential biomarker for the diagnosis and detection of premalignant gastric cardia lesions and early-stage GCA. By identifying the gene mutation, it may be possible to detect potential premalignant cardia lesions at an earlier stage. This early detection can facilitate prompt treatment, leading to a reduction in the disease burden associated with GCA.

## Author contributions

**Conceptualization:** Guangda Wang, Limian Er.

**Data curation:** Guangda Wang, Yan Lin.

**Formal analysis:** Guangda Wang, Liang Liu.

**Methodology:** Guangda Wang, Liang Liu, Yang Zhao.

**Project administration:** Limian Er.

**Resources:** Liang Liu, Yang Zhao, Yan Lin.

**Software:** Liang Liu, Yan Lin.

**Supervision:** Guangda Wang, Limian Er.

**Visualization:** Guangda Wang, Yang Zhao.

**Writing – original draft:** Guangda Wang, Liang Liu, Yang Zhao, Yan Lin.

**Writing – review & editing:** Guangda Wang, Limian Er.

## Supplementary Material


