# Supplementary figures and images for: Comparative genomic analysis unveiling the mutational landscape associated with premalignant lesions and early-stage gastric cardia cancer
Source: Medicine (Baltimore). 2025 Jan 10;104(2):e40332. doi: 10.1097/MD.0000000000040332 (PMC11731115; doi:10.1097/MD.0000000000040332)

Supplementary Figure 1

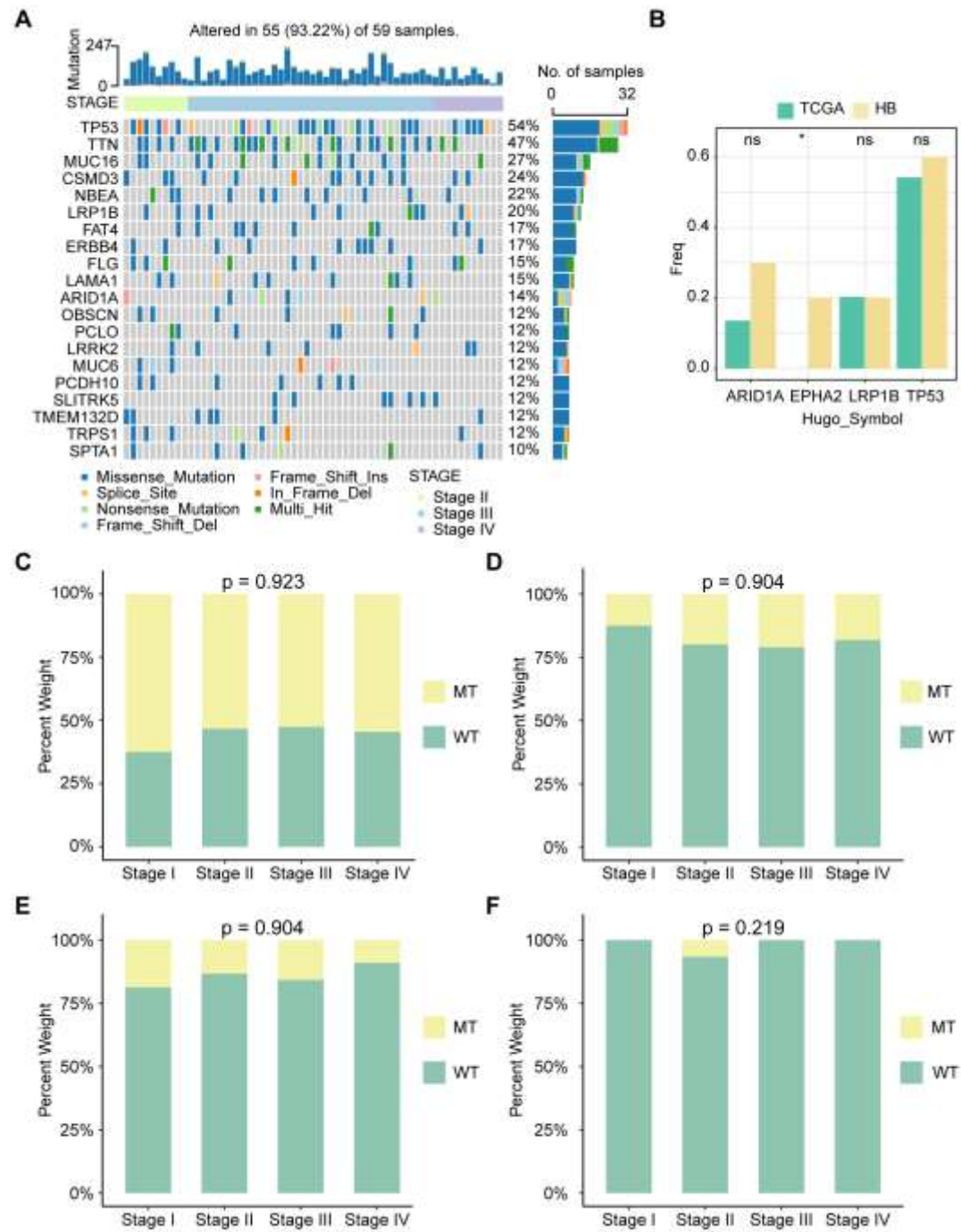

Supplementary Figure 2

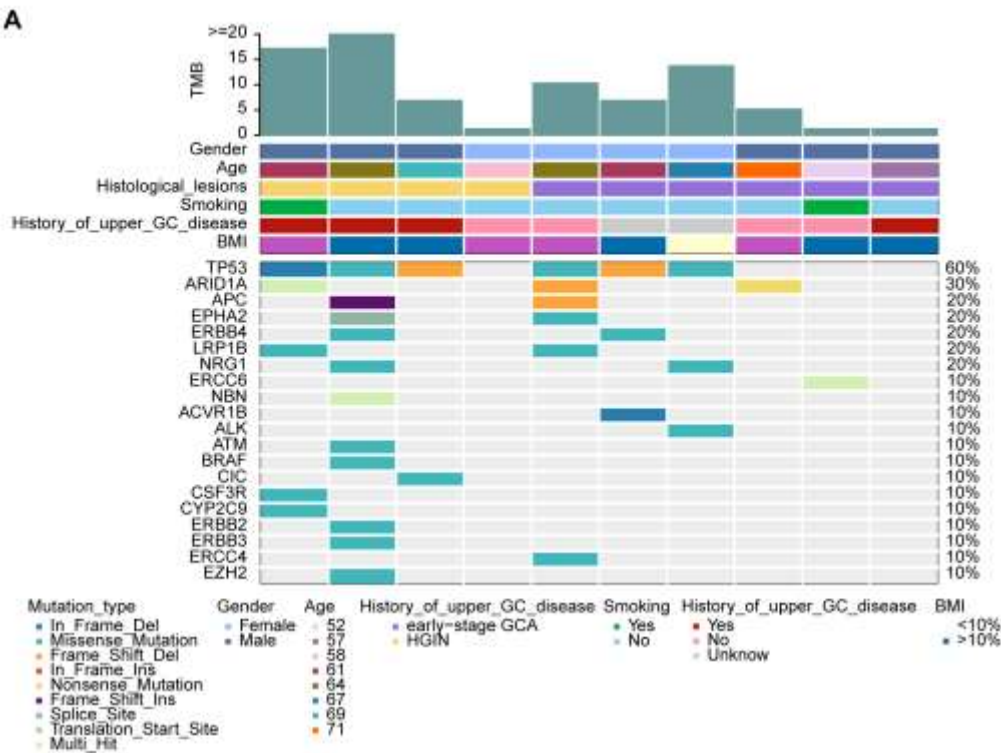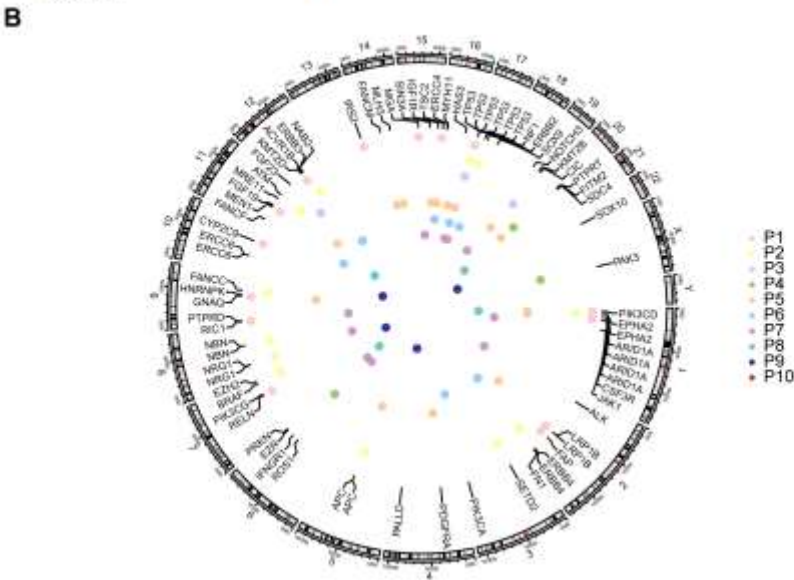

Supplementary Figure 3

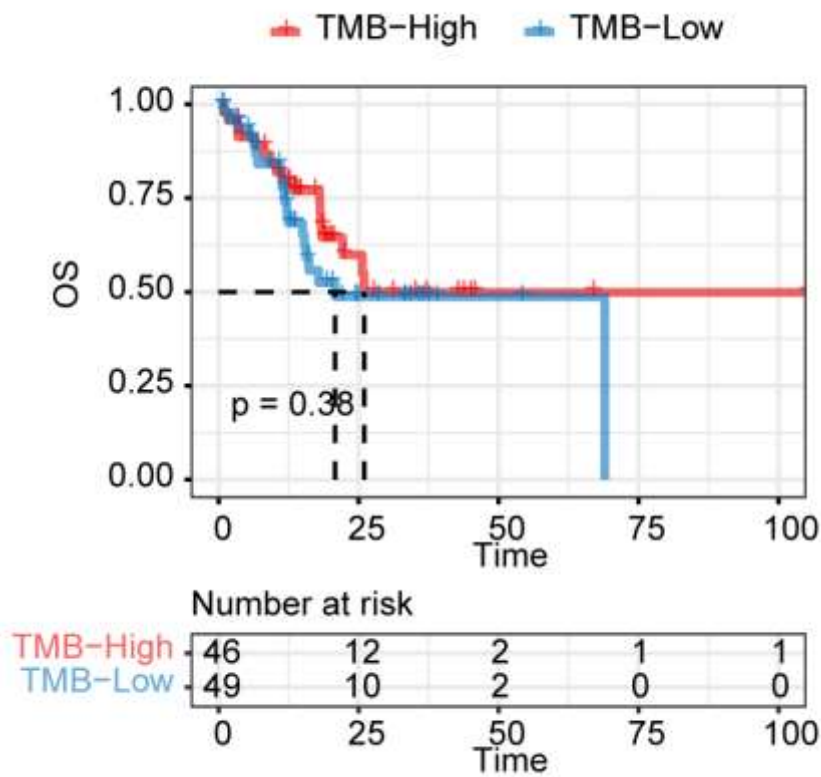

Supplementary Figure 4

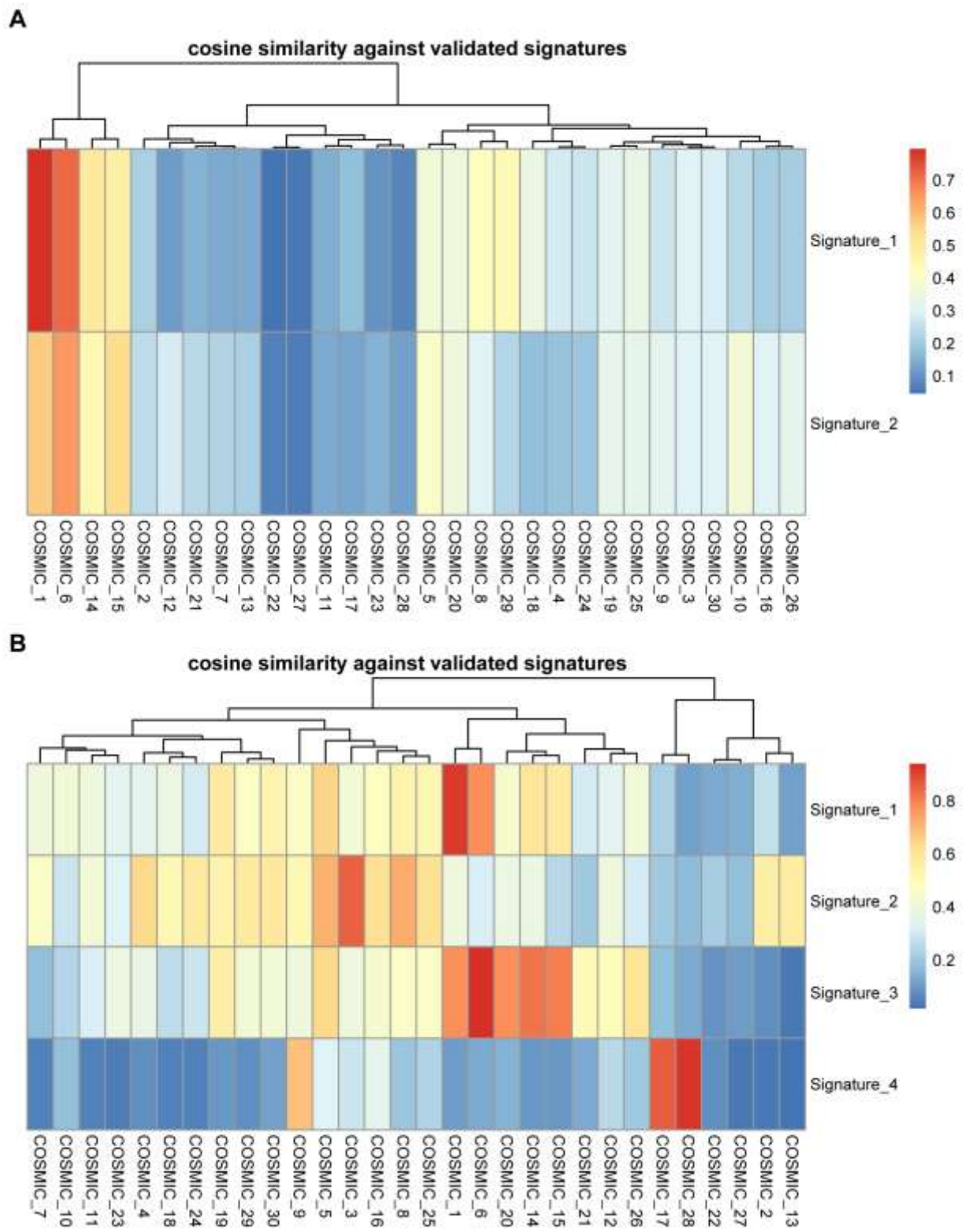

Supplement: Supplementary file 1 [file medi-104-e40332-s001.pdf]
